# Primary Intraosseous Osteolytic Meningioma with Aggressive Clinical Behaviour: Clinico-Pathologic Correlation and Proposed New Clinical Classification

**DOI:** 10.3390/life12040548

**Published:** 2022-04-06

**Authors:** Nazmin Ahmed, Gianluca Ferini, Moududul Haque, Giuseppe Emmanuele Umana, Gianluca Scalia, Bipin Chaurasia, Atul Vats, Asifur Rahman

**Affiliations:** 1 Department of Neurosurgery, Ibrahim Cardiac Hospital and Research Institute (A Centre for Cardiovascular, Neuroscience and Organ Transplant Units), Shahbag, Dhaka 1000, Bangladesh; nazmin.bsmmu@gmail.com; 2 Department of Radiation Oncology, REM Radioterapia srl, 95029 Catania, Italy; gianluca.ferini@grupposamed.com; 3 Department of Neurosurgery, Bangabandhu Sheikh Mujib Medical University, Shahbag, Dhaka 1000, Bangladesh; moududneurosurg@gmail.com (M.H.); bijoun14@yahoo.com (A.R.); 4 Trauma Center, Gamma Knife Center, Department of Neurosurgery, Cannizzaro Hospital, 95100 Catania, Italy; 5 Department of Neurosurgery, Highly Specialized Hospital and of National Importance “Garibaldi”, 95126 Catania, Italy; gianluca.scalia@outlook.it; 6 Department of Neurosurgery, Neurosurgery Clinic, Birgunj 44300, Nepal; trozexa@gmail.com; 7 Neurosurgery Department, James Cook University Hospital, Middlesbrough TS4 3BW, UK; vatsatul7@gmail.com

**Keywords:** PIOM, pathology, treatment, surgery, classification

## Abstract

(1) Introduction: Primary intraosseous osteolytic meningiomas (PIOM) are non-dural-based tumors predominantly presenting an osteolytic component with or without hyperostotic reactions. They are a subset of primary extradural meningiomas (PEM). In this study, we present a peculiar case with a systematic literature review and propose a new classification considering the limitations of previous classification systems. (2) Materials and Methods: Using a systematic search protocol in Google Scholar, PubMed, and Scopus databases, we extracted all case studies on PIOM published from inception to December 2020. A 46-year-old female patient form Dhaka, Bangladesh, was also described. The search protocol was performed according to the Preferred Reporting Items for Systematic Reviews and Meta-Analyses (PRISMA) guidelines. (3) Results: Here, we present a 46-year-old female patient with PIOM who successfully underwent bifrontal craniotomy and gross total removal (GTR) of the tumor. At 6-month follow-up, no tumor recurrence was shown. Including our new case, 55 total cases from 47 articles were included in the analysis. PIOMs were in closer frequency among males (56.4%) and females (43.6%). The most common tumor location was the frontal and parietal calvarium, most commonly in the frontal bone (29.1%). Surgical resection was the predominant modality of treatment (87.3%); only 1.8% of patients were treated with radiotherapy, and 5.4% received a combination of surgery and radiotherapy. Gross total resection (GTR) was achieved in 80% of cases. Extracranial extension was reported in 41.8% of cases, dural invasion in 47.3%, and recurrence in 7.3%. Whole-body 68 Ga-DOTATOC PET/CT has also been reported as a useful tool both for differential diagnosis, radiotherapy contouring, and follow-up. Current treatments such as hydroxyurea and bevacizumab have variable success rates. We have also suggested a new classification which would provide a simple common ground for further research in this field. (4) Conclusions: Surgical resection, especially GTR, is the treatment of choice for PIOM, with a high GTR rate and low risk of complications and mortality. More research is needed on the differential diagnosis and specific treatment of PIOM.

## 1. Introduction

Meningiomas are typically slow-growing tumors that arise from arachnoid cap cells [1]. Meningiomas are the most common primary CNS tumor and were well described in the centuries before Harvey Cushing coined the term in 1922 [2]. They represent 37.6% of all primary brain tumors in adults, making them the most common type of intracranial tumor with an incidence of 8.83 per 100,000 in the most recent Central Brain Tumor Registry of the United States [3]. Risk factors include exposure to ionizing radiation such as during radiation therapy, a familial predisposition, and neurofibromatosis type 2 [4,5]. In contrast, primary intraosseous meningioma (PIOM) is a term used to describe a subset of extradural meningiomas that arise in bone. They represent a subtype of primary extradural meningiomas (PEM), a relatively rare entity accounting for less than 2% of all meningiomas [6,7]. They may arise from other locations, such as the skin, orbit, nasopharynx, and neck [8,9]. It represents approximately two-thirds of all extradural meningiomas [10]. Especially, among all PIOMs, PIOMs with both osteolytic radiological features and atypical pathological features are extremely rare. In addition, there are few reports about dural involvement of the PIOM [9,11,12,13]. PIOMs are usually mistaken for primary bone tumors and appear more prone to develop malignant features compared to intracranial meningiomas [10,14,15] Preoperatively diagnosing a scalp mass as an intraosseous meningioma is challenging, particularly when it is on both the calvarium and the scalp. Typical meningiomas appear as dural-based lesions isointense to gray matter on both T1- and T2-weighted magnetic resonance imaging (MRI) and are contrast-enhanced on both MRI and computed tomography (CT). As in the case described here, preoperative diagnosis of an intraosseous meningioma of the skull is difficult if imaging shows osteolysis of the inner and outer plates of the skull [14]. Recently, 68Ga-DOTATOC PET/CT has been suggested as a useful tool for the radiological confirmation of meningioma, essential for upfront gamma-knife procedures, as well as during follow-up after GK [15,16,17,18] PIOMs are very rare, and because of their rarity, their epidemiology, natural history, clinical presentation, differential diagnosis from neuroimaging, optimal surgical strategy, and outcome are described in different case reports and series in a scattered manner. Thereby, a thorough systematic review is mandatory to understand the disease process and timely intervention to achieve optimal outcomes. We present here a systematic review of PIOMs with special emphasis on their pathogenesis, mechanism of osteolytic reaction, preferred location, clinical features, diagnosis, treatment, and future research. In addition, we propose a new classification system considering the limitations of previous classifications.

## 2. Materials and Methods

### 2.1. Search Strategy

**Figure 1:**
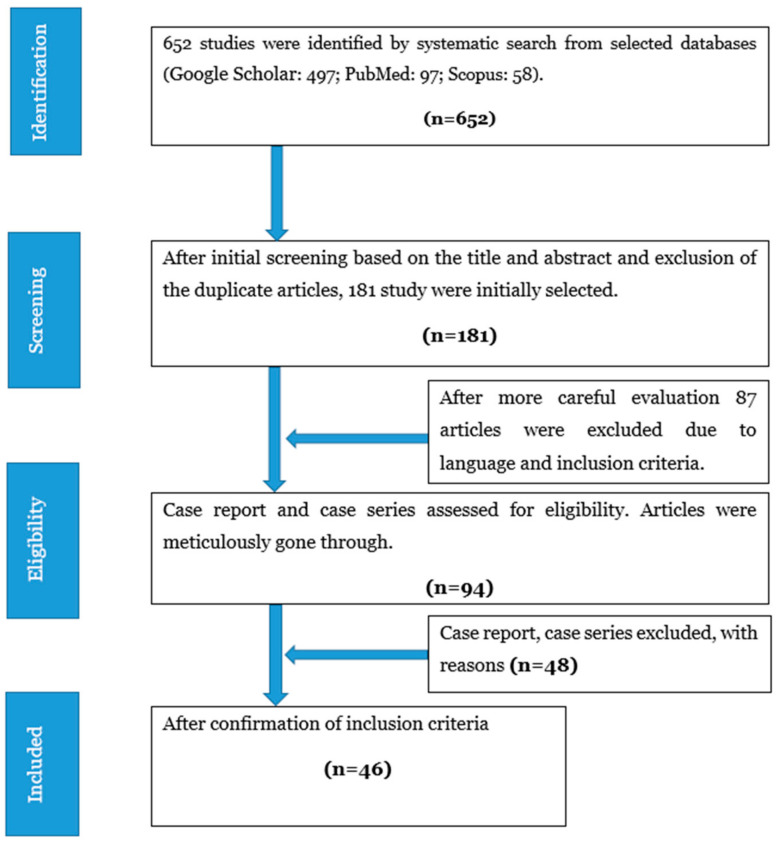
PRISMA flow diagram for study selection.

We searched Google Scholar, PubMed, and Scopus databases for the selection of peer-reviewed published articles for our systematic review with appropriate mesh terms. Only case reports and case series of PIOM were found. Therefore, during the selection procedure, we screened published case reports and case series from the inception to December 2020 following the search criteria. We restricted the screening language to only English. The search terms included “primary intraosseous meningioma”, “primary intraosseous osteolytic meningioma”, and “PIOM” to incorporate all potential articles in our analysis. The Mendeley citation manager was used for the management of the articles collected through our systematic search (Figure 1). The study is in line with the Preferred Reporting Items for Systematic Review and Meta-Analysis (PRISMA) guidelines.

### 2.2. Selection Criteria

To meet the objectives of our study, we included all available case reports and case series regarding PIOM involving the skull vault and base and reviewed them meticulously. Papers lacking necessary information regarding demographic characteristics, clinical presentation, diagnostic modalities, treatment, histopathology, and outcome were excluded.

### 2.3. Data Analysis

The information from selected research articles were recorded in Microsoft Excel 2013. We further reviewed the articles for missing information and checked for consistency. Data analyses were conducted by IBM SPSS (version-23) statistical package software. (Table 1) 

### 2.4. Case Description

#### 2.4.1. Clinical History

A 46-year-old female patient was admitted to the Neurosurgery Outpatient Department of Ibrahim Cardiac Hospital and Research Institute, Dhaka, Bangladesh, in 2019, complaining of a large subcutaneous mass in the frontal area. She first noticed a small, non-tender, hard lump in the mentioned area 8 years ago. The lesion increased very slowly over time. Two years ago, she presented a papillary carcinoma of thyroid and underwent total thyroidectomy. Due to the presence of the lesion, there was clinical suspicion of skull metastasis. As the patient denied any neurosurgical intervention, she was advised to receive whole-brain radiotherapy. After completion of radiotherapy, she noticed rapid enlargement of swelling, along with headaches. For the past 8 months, due to additional changes in her personality and behavior, she had an MRI of her brain and was referred to our department for further evaluation and management.

#### 2.4.2. Physical Examination

Local examination of the mass demonstrated bony, hard, mildly tender swelling of 6 cm × 4 cm × 3 cm in the frontal region. The mass had ill-defined margins with an irregular surface, fixed with overlying skin as well as underlying structures. There were no palpable lymph nodes and no swelling elsewhere in the body. Metastatic work-up was negative.

#### 2.4.3. Preoperative Imaging

**Figure 2:**
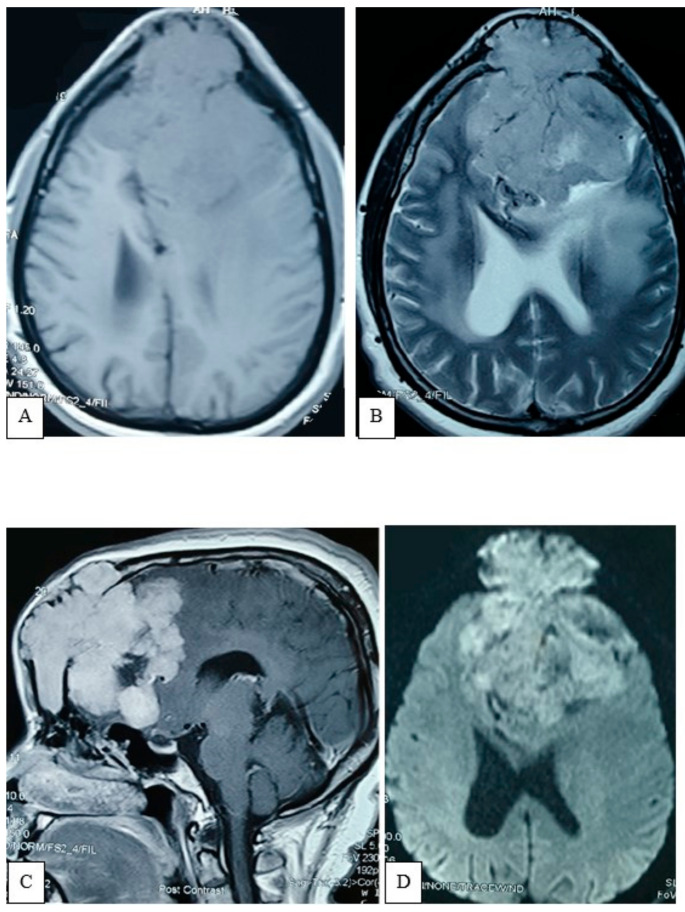
Brain MRI demonstrates a T1WI iso to hypointense (**A**) and T2WI heterogeneously hyperintense (**B**) mass present in both frontal regions, having extra-calvarial and intradural extension and invasion of the brain parenchyma. Moderate perilesional edema and gross midline shifting are seen. Post-contrast, sagittal (**C**) and DWI (**D**) section demonstrates moderate heterogenous contrast enhancement with central non enhancing area, representing necrosis. Broad base attachment lies within the diploic space. Mass effect is evident by the compression over corpus callosum and frontal horn of both lateral ventricles. Restricted diffusion present in scattered areas within the tumor.

**Figure 3:**
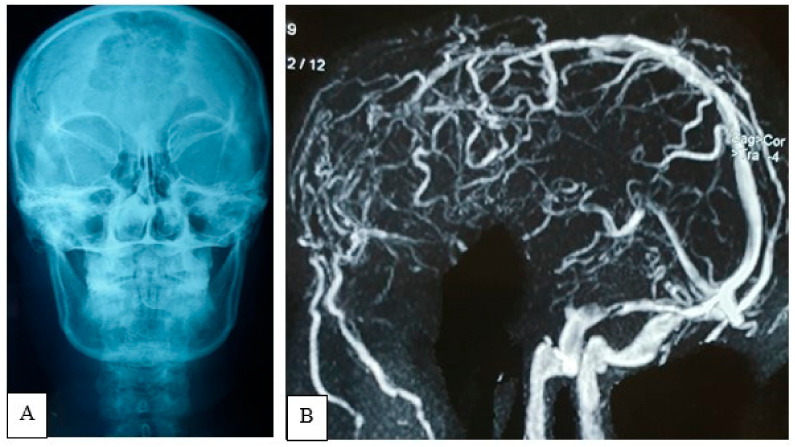
Plain X-ray of the skull, AP view (**A**) showing expansile lytic lesion with internal septation is noted within frontal bone. MRV (**B**) oblique view demonstrates anterior third of the SSS obliterated with multiple aberrant collaterals.

A plain X-ray of the skull showed an expansile lytic lesion having internal septations located in the frontal bone, with a bulging of overlying soft tissue shadow. There was no abnormal calcification. Vascular markings appeared to be normal; all the features were suggestive of metastasis. For better delineation of the pathology, an MRV was also performed. There was an irregular, lobulated extra-axial T1WI iso to hypointense and T2WI heterogeneously hyperintense mass measuring about 7.9 cm × 7.6 cm × 7.4 cm noted in both frontal regions (Figure 2). Mass effect was evident by compression and displacement over both frontal lobes, sub-falcine herniation, and compression over genu and body of corpus callosum and lateral ventricles. The mass was causing destruction of the overlying frontal bone and extending into the subcutaneous region. After intravenous contrast administration, moderate heterogenous enhancement of the lesion was observed with a central non-enhancing area, representing necrosis. MRV, post-contrast sequence, showed obliteration of the anterior third of superior sagittal sinus with multiple dilated collateral vascular channels (Figure 3).

#### 2.4.4. Surgical Procedure

**Figure 4:**
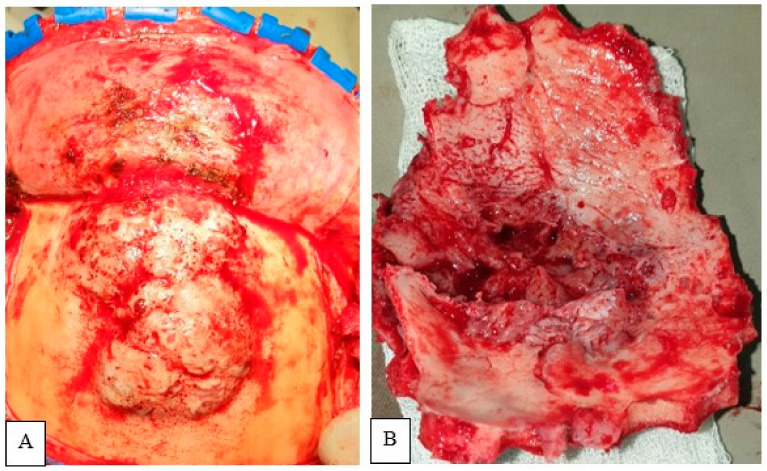
Intraoperative photograph showing evidence osteolysis with infiltration of overlying subcutaneous tissue (**A**,**B**).

The tumor was exposed through a bicoronal incision and subgaleal dissection. The mass presented diffuse infiltration of the subcutaneous tissue. After meticulous dissection, the flap was retracted antero-inferiorly (Figure 4). A bifrontal craniectomy was performed. Bone was eroded and its intracranial counterpart identified. The tumor showed both extracranial and intracranial extension, with a centrally placed dural defect. The mechanical compression of the tumor might result in this dural defect. Frontal sinus was occupied by the tumor tissue. With microsurgical technique, the intracranial soft tissue part was removed in a piecemeal fashion. There was infiltration of the brain parenchyma, which was meticulously dealt with. GTR of the tumor was accomplished. After careful hemostasis, duroplasty with G-patch followed by cranioplasty with polymethyl methacrylate concluded the surgical procedure.

#### 2.4.5. Post-Operative Course

**Figure 5:**
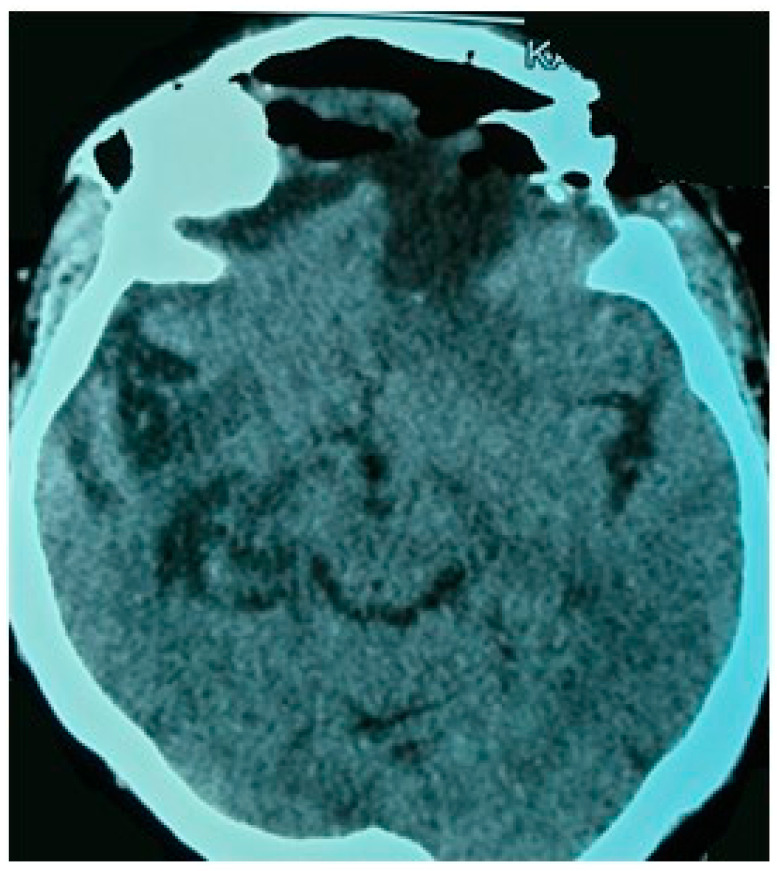
Post-operative brain CT scan: axial section demonstrates gross total resection of tumor.

The patient presented an uneventful recovery. There was no onset of new neurological deficits during follow-up. Post-operative head CT scan documented a complete tumor removal (Figure 5). She was clinically stable at 6 months follow-up.

#### 2.4.6. Histopathology

**Figure 6:**
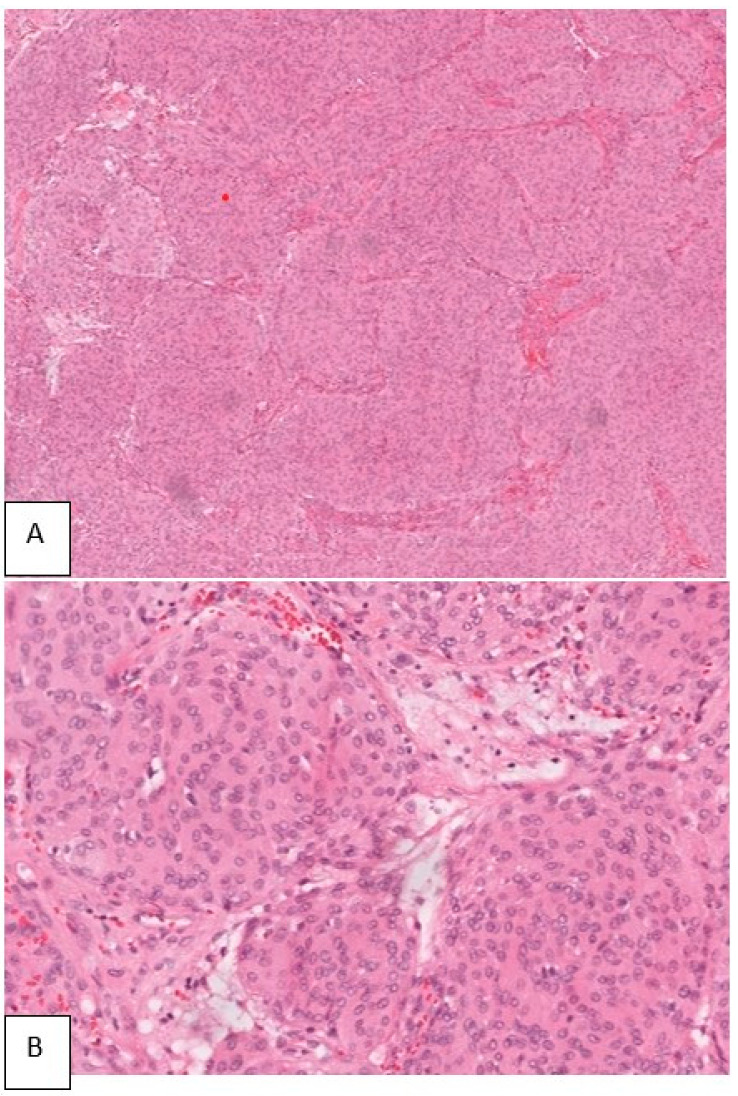
Photomicrograph of the biopsy specimen showing the tumor cells arranged in lobular configuration (H&E 40×) (**A**). Cells having round nuclei with ill-defined cytoplasm. Infiltration of surrounding bone present (H&E 100×) (**B**).

Sections made from the submitted specimen show a meningothelial meningioma with whorl formation. The cells were epithelioid in shape, having oval nuclei. No mitosis or necrosis were seen (Figure 6).

## 3. Results

We presented a 46-year-old female patient treated for PIOM. Her follow-up head CT scan showed GTR, and the patient was clinically stable at 6 months. Including our new case, a total of 55 cases from 47 articles were considered for the analysis. The mean age of the study participants was 55.38 (range 6–84 years); 31 out of 55 (56.4%) were males and 24 out of 55 (43.5%) were females. The most common tumor location was the frontal and parietal calvarium, with the frontal bone being the most common occurrence (29.1%) of the cases, the parietal bone in 23.6%, and a combination of the frontoparietal bone in 10.9% of the cases. The most common symptom was a visible mass lesion, which occurred in 52.7% of the patients, and it was typically a growing mass. Surgical resection alone was the predominant modality of treatment, occurring in 87.3% of the cases. Only 1.8% of patients were treated with radiation alone, and 5.4% received a combination of surgery and radiation. Gross total resection was achieved in 80% of cases. The mean post-operative follow-up interval was 15 months. Extracranial extension was reported in 41.8% of cases and dural invasion was reported in 47.3% of cases. We categorized the PIOMs according to the histopathology following the WHO categories type I (74.5%), type II (16.4%), and type III (9.1%). Recurrence was reported in 7.3% of the patients.

## 4. Discussion

### 4.1. Classification of PIOM

PIOM is a term used to describe a subset of primary extradural meningioma that arise in bone, when no dural attachment is present. They can present either as an osteoblastic lesion or an osteolytic lesion [59]. The term “intraosseous” was used to describe those meningiomas limited to skull bones with no epidural or subcutaneous components [60]. They are special subset of PEM, which has been classified by Lang et al. into three types, depending on their origin and the extent of extracranial and intracranial soft tissue involvement. These are purely extra-calvarial (type I), purely calvarial (type II), and calvarial with extracalvarial extension (type III) [9].

### 4.2. Mechanism of Osteolysis

There are scant literature addressing the mechanism of osteolysis in PIOM. In 2007, Sade et al. showed integrin-mediated adhesion of osteoclasts to the bone matrix in the case of skull base meningioma, which promotes degradation of bone collagen by releasing lysosomal enzymes (ITG B1) [61]. Moreover, Salehi et al. demonstrated higher levels of OPN and ITG B1 expression in tumor vasculature, suggesting a vascular-dependent role. Other studies focus on the role of MMP 2 with respect to brain invasion, peritumoral edema, and tumor recurrence [62]. However, the findings are still now a matter of debate.

### 4.3. Incidence

Although meningiomas are the most common extra-axial tumor in adults, intraosseous meningiomas are rare tumors that originate in the skull, accounting for 1–2% of all meningiomas [6]. The majority of meningiomas are intradural, whereas primary extradural meningiomas (PEMs) originate outside the dural layer of any part of the brain or spinal cord and do not have any connection to the dura mater or any intracranial structures [9]. Hoye et al. emphasized that ectopic meningiomas do not have any connection with the foramina of any cranial nerves or with any intracranial structures [63]. On the other hand, other reports demonstrated that PEM could show intracranial growth involving the dura mater. Bassiouni et al. suggested that 14 of 16 (88%) PEM patients who underwent surgery had a true dural involvement, which was proven [41]. In another report, the inner and outer dura seemed to be uninvolved by the tumor in the intraoperative finding, but a tumor infiltration to inner and outer dura was pathologically proven [64]. Thus, PIOM with “dural involvement” can cause ambiguity regarding PEM, and the exact definition is yet to be disclosed.

### 4.4. Clinical Presentation

Intraosseous meningiomas usually occur in both males and females with the same frequency or with a slight predominance among females [41]. However, our analysis suggests dominance in males. They predominantly occur later in life, with a median patient age at diagnosis in the fifth decade, as suggested by the findings of our analysis [9]. In our study, the most common symptom was a palpable mass lesion, which occurred in 52.7% of the patients, and it was typically a growing mass. According to previously conducted studies, the majority of intraosseous meningiomas in the base of the skull and orbit are usually asymptomatic, but may present pain, proptosis, and neurological symptoms [13].

### 4.5. Neuroimaging Features and Differential Diagnosis

According to the literature, hyperostosis is present in 59% of PIOM imaging evidence, osteolytic changes in the surrounding bone appear in 32% of cases, and mixed features of osteolysis-hyperostosis are reported in 6% of cases [14]. The bone expansion and hyperdense skull lesions may appear radiologically, e.g., en plaque meningioma, osteoma, osteosarcoma, Paget’s disease, and fibrous dysplasia [65]. PIOMs with osteolytic skull lesions may rarely show as hypodense bone feature outlined by a hypodense border zone [66]. These PIOMs with an osteolytic radiographic appearance may occur with a malignant behavior (progress rapidly and invade the surrounding structures) and show malignant or anaplastic histopathology [66,67]. The differential diagnoses of osteolytic meningioma include metastasis and sarcoma. CTs show osteolytic hypodense lesions in metastatic conditions that thin the calvarium and erode through the inner or outer tables of the skull, sometimes associated with soft-tissue mass. Metastatic lesions or sarcoma might progress more rapidly than meningioma, but it is difficult to make the diagnosis in this subtype before operation and biopsy [31]. 68Ga-DOTATOC PET/CT has been reported to represent a useful tool for differential diagnosis, and during follow-up to detect possible tumor recurrence. Other possible uses of 68Ga-DOTATOC PET/CT include tumor contouring for radiotherapy RT planning and subsequent follow-up in which SUV modification can suggest tumor control after RT [15,68]. Whole-body 68 Ga-DOTATOC PET/CT has also been reported to detect incidentalomas and/or extracranial meningiomas [15].

### 4.6. Extent of Dura and Soft Tissue Involvement

A review conducted by Lang et al. identified dural involvement in 60% (CT or MR imaging) of PIM patients. On visual inspection after craniotomy, the dura appeared normal in 40% of the cases [9]. According to our analysis, extracranial extension was reported in 41.8% of cases and dural invasion was reported in 47.3% of cases. However, Bassiouni et al. reported that 88% of patients had a true dural involvement in PEMs of the cranial vault [41]. In addition, dural involvement of the PIM can be represented with the “dural tail sign” radiologically. Although the dural tail sign generally was first thought to be pathognomonic of meningioma of the dural origin, it can also be presented by pituitary adenomas, schwannomas, and astrocytomas [9,69]. Yamazaki et al. concluded that PIMs do not involve the underlying dura. If the dura is involved, it is suggestive for secondary invasion of the bone [38]. After our literature review, we suggest that PIOM generally has more tendency to form a broader base in the calvarium than in the dura, while tumors of meningeal origin including meningioma have a broader base in the dura than in the calvarium.

### 4.7. Recommended Management Strategy

Surgical resection (GTR) is the major treatment of choice for primary intraosseous meningiomas, with low risk of complications and mortality reported. When feasible, wide en bloc resection including 1 cm negative margins is recommended in high-grade meningioma [70]. In the study by Bassiouni et al [41]., the unexpectedly high recurrence rate of 13.3% in tumors with benign histological features corresponds with that of 22% reported by Lang et al [9]. and presumably due to the presence of microscopic islands of neoplasm persisting in the dura which, at the macroscopic level, had a normal appearance. Therefore, we suggest the removal of dura at the site of bone involvement and the subsequent undertaking of pathological assessment. Wide surgical excision is the main treatment for extradural meningiomas, and it is potentially curative if complete resection is achieved [70]. Current treatments are targeting molecular pathways in the treatment of meningiomas, such as hydroxyurea and bevacizumab, with variable success rates [71]. However, more research is needed for the specific treatment of PIOM. Previously, the calvarial defects were reconstructed with artificial bone material such as polymethyl methacrylate. Now, custom-made 3D cranial prostheses are used for their reliability, less time consumption, and reasonable cost. Custom-made 3D cranial prostheses are also favorable in terms of their aesthetic, functional outcomes, and fewer complications [72,73].

### 4.8. Outcome

In our analysis, recurrence was noted in 7.3% of cases, which is lower than another previously conducted study, where recurrence was noted in 22% of cases of benign PEMs [41]. On the other hand, a recurrence rate of 33% was reported in cases of tumors with atypical or malignant histological features [41]. Partington et al. reported that carcinoembryonic antigen (CEA), which is an oncofetal glycoprotein, is associated with atypical meningioma without secretory features, and a decline in CEA levels is associated with effective treatment of the symptomatic tumor [10].

### 4.9. Proposed New Classification

**Figure 7:**
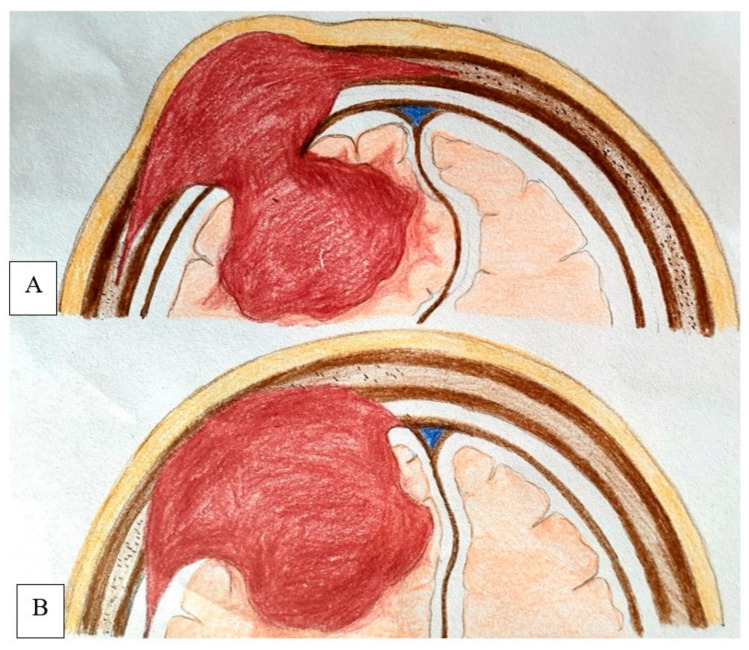
Schematic illustration demonstrates the mode of intracranial extension in PIOM, where the tumor broad base lies within the diploic space, erodes the dura, and invades the brain parenchyma (**A**) and typical convexity meningioma with extension into overlying bone whereas the broad base lies along the dura (**B**).

The classification by Lang et al. was simple and useful from a topographical point of view, but it has some limitations [9]. Some tumor subgroups presented in case reports cannot be classified using the Lang Scale [41]. Some meningiomas are located between the dura mater and the inner calvarial table, primary cutaneous meningiomas, and extracalvarial meningiomas attached to the outer calvarial table [74,75]. In published cases, the inner table was disrupted in 73% of calvarial meningiomas, and some of these tumors abutted the dura. Additionally, some cutaneous meningiomas were connected to the dura through an osseous defect by a connective tissue stalk, which was shown histologically to contain tumor cellsNone of the previous classification systems considered a tumor’s involvement in the dura mater. Therefore, we included type IV (mixed variety), defined as tumors extending from the dura to the extracalvarial space. Based on the pertinent literature and on our own experience, we suggest the use of this classification, which takes these differences into account (Table 2) and provides a simple common ground for further research in this field. This concept is demonstrated by a schematic illustration in Figure 7.

## 5. Conclusions

This study revealed a new case of PIOM in Bangladesh which successfully underwent bifrontal craniotomy and gross total removal (GTR). Based on our analysis, we recommend complete resection as the treatment of choice for these PIOMs. Serial follow-up to confirm recurrence or progression should be conducted after the surgery. 68 Ga-DOTATOC PET/CT is a useful tool for differential diagnosis, RT contouring, and follow-up. The study also revealed a new classification which would assist researchers and clinicians in further research in this field and in decision making. More research is required on the mechanism of osteolysis, management strategies, and specific treatment. 

## Figures and Tables

**Table 1:** Existing cases of primary intraosseous osteolytic meningioma (PIOM).

Author	Year	Age	Sex	Location	Clinical Presentation	Scalp Mass	Extracranial Extension	Dural Invasion	Mx	WHO Grade (Hist)	Outcome
**Klein et al.** [19]	1975	66	F	P	Scalp mass	yes	yes	yes	GTR	1	Not mentioned
**McWhorter et al.** [20]	1976	42	M	T	Scalp mass	yes	yes	no	GTR	1	Not mentioned
**Palma et al.** [21]	1977	18	M	Fr	Intracranial hypertension	no	no	no	NA	1	Not mentioned
**Pearl et al.** [22]	1979	44	F	Fr	Headache, dizziness	no	no	no	GTR	1	No recurrence at 3 months F/U CT
**Ohaegbulam** [23]	1979	31	M	Fr	Scalp mass	yes	no	yes	NA	1	NA
**Young** [24]	1983	71	M	Fr	Scalp mass	yes	no	no	GTR	1	No recurrence at 6 months
**Lee et al.** [25]	1988	61	M	Fr, T	Scalp mass	yes	yes	yes	GTR, RT	3	Recurrence after 2 years, no metastasis
**Kaneko et al.** [26]	1988	71	F	FP	Scalp mass	yes	no	yes	GTR	1	NA
**Oka et al.** [27]	1989	79	F	FP	Scalp mass	yes	yes	no	GTR	1	No recurrence after 4 year and 9 months
**Ammirati et al.** [28]	1990	21	M	T	Facial weakness	no	yes	yes	GTR	1	No recurrence at 13 months F/U
**Kulali et al.** [29]	1991	50	M	O	Scalp mass	yes	no	no	GTR	1	No recurrence at 2 years F/U
**Ito et al.** [30]	1992	72	F	FP	Scalp mass	yes	no	no	GTR	1	Not mentioned
**Fujita et al.** [31]	1993	42	M	TP	Facial weakness, hearing difficulty	no	yes	yes	STR, RT	3	Patient died after 1 year due to respiratory failure following metastasis
**Ghobashy and Tobler** [32]	1994	65	F	Fr	Headache	no	no	no	GTR	1	No recurrence at 2 years F/U
**Partington et al.** [11]	1995	84	F	FT	Scalp mass, aphasia	yes	yes	yes	GTR, RT	2	No recurrence after 8 months
**Kuzeyli et al.** [33]	1996	6	M	T	Scalp mass	yes	no	no	GTR	1	Not mentioned
**Changhong et al.** [34]	1997	42	F	O	Scalp mass	yes	no	no	NM	3	Not mentioned
**Muthukumar** [35]	1997	55	M	P	Scalp mass	yes	yes	no	GTR	1	Not mentioned
		50	M	TP	Personality change, aphasia	no	yes	no	GTR	1	Patient lost F/U
		65	M	Fr	Scalp mass	yes	yes	no	GTR	1	Not mentioned
**Kudo et al.** [36]	1998	56	F	P	Vertigo	no	no	yes	GTR	1	Not mentioned
**Okamoto et al.** [37]	2000	78	F	P	H/A	no	no	no	GTR	1	No recurrence after 18 months F/U
**Lang et al.** [9]	2000	59	M	SW	Scalp mass	yes	yes	yes	GTR	2	Not mentioned
**Yamazaki et al.** [38]	2001	62	M	O	Vomiting, nystagmus, dysmetria	no	no	yes	GTR	1	No recurrence after 18 months F/U
**Rosahl et al.** [39]	2004	38	M	T	Acute hearing loss	no	no	no	GTR	1	Uneventful recovery
**Tokgoz et al.** [40]	2005	44	M	FT	Scalp mass	yes	yes	no	GTR	2	No recurrence after 1 year F/U
**Bassiouni et al.** [41]	2006	62	F	Fr	Not mentioned	no	no	yes	GTR	2	Not mentioned
	2006	47	M	P	Not mentioned	no	no	yes	GTR	1	Not mentioned
	2006	46	F	T	Not mentioned	no	no	no	GTR	1	Recurrence after 3 years F/U
	2006	34	M	T	Not mentioned	no	yes	yes	GTR	1	Not mentioned
	2006	57	F	P	Not mentioned	no	no	yes	GTR	1	Not mentioned
**Agrawal et al.** [42]	2007	70	F	Fr	Scalp mass	yes	no	yes	NTR	1	No evidence of resurrection after 4 months, dural enhancement recorded
**Al-Khawaja et al.** [43]	2007	50	M	P	H/A, scalp mass	yes	no	yes	GTR	1	Uneventful recovery
**Sheikhrezaie et al.** [44]	2009	62	M	FP	Scalp mass	yes	no	no	GTR	1	Not mentioned
**Yener et al.** [45]	2009	78	M	P	Asymptomatic	no	no	no	GTR	1	Uneventful recovery
**Hong et al.** [46]	2010	52	M	P	Asymptomatic	no	no	no	GTR	1	No residual at post-operative CT scan
	2010	73	M	O	Scalp mass	yes	no	no	GTR	3	No residual at post-operative CT scan
**Yilmaz et al.** [47]	2010	41	M	Fr	Scalp mass	yes	no	yes	GTR	1	Not mentioned
**Kim et al.** [12]	2012	68	M	P	Scalp mass	yes	yes	yes	GTR	2	Recurrence at multiple sites of whole skull after 1 year F/U
		74	F	Fr	Scalp mass	yes	yes	yes	GTR	3	Recurrence after 19 months and 45 months F/U, underwent 2 times surgery. After 5 years, documented metastasis.
**Akhaddar and Ennouali** [48]	2014	37	F	Fr	Headache, scalp mass	yes	yes	no	GTR	1	No recurrence after 1 year F/U
**Tang et al.** [49]	2014	82	F	P	Gait difficulty, memory impairment	no	no	no	Biopsy	1	Not mentioned
**Yun and Lee** [10]	2014	65	F	Fr	Scalp mass	yes	yes	yes	GTR	2	No recurrence after 6 months F/U
**Kim et al.** [50]	2014	44	F	SW	Headache, proptosis	no	yes	no	GTR	1	No recurrence after 6 months F/U
**Bujok and Bienioszek** [51]	2014	59	F	Fr	Headache, memory impairment	no	no	yes	GTR	1	Uneventful recovery
**Kwon et al.** [6]	2015	69	M	P	Scalp mass, headache	yes	yes	yes	GTR	1	No recurrence after 6 months F/U
**Hong et al.** [52]	2015	61	M	FP	Headache, upper limb weakness	no	no	no	GTR	1	No recurrence after 1 month F/U
**Ben Nsir et al.** [53]	2016	42	M	T	Hearing difficulty, facial asymmetry, vertigo	no	yes	no	IMRT	2	No new deficit after 8 months F/U
**Bohara et al.** [54]	2016	38	M	P	Scalp mass	yes	yes	yes	GTR	2	No recurrence after 6 months F/U
**Mouri et al.** [55]	2017	76	F	Fr	Dizziness	no	no	yes	GTR	1	Uneventful recovery
**Richardson et al.** [56]	2017	23	M	Fr	Scalp mass	yes	no	no	GTR	1	No recurrence after 2 years F/U
**Kwon et al.** [6]	2019	80	M	T, O	Hearing loss, dizziness, balance difficulty	no	yes	yes	NTR	2	Uneventful recovery
**Abuzayed et al.** [57]	2019	78	F	Clivus	Vertigo, diplopia	no	no	no	NTR	1	Uneventful recovery
**Echchikhi Meryem et al.** [58]	2020	60	F	Fr	Headache, bulge	no	no	no	GTR	1	No post-operative complications
**Present case**	2020	46	F	FP	Scalp mass, personality change	yes	yes	yes	GTR	1	No recurrence after 6 months F/U

**Table 2:** Classification of primary intraosseous meningiomas.

Types	Description
Type I	PIM restricted within diploic space, having osteoblastic or osteolytic or mixed reaction
Type II	PIM outweigh the diploic boundary, having extracranial or intracranial component with displacement of the surrounding structures
Type III	PIM outweigh the diploic boundary, having extracranial or intracranial component with invasion of the surrounding structure
Type IV	Any of the above criteria with documented features of metastasis
